# Rapid-Rate Paired Associative Stimulation over the Primary Somatosensory Cortex

**DOI:** 10.1371/journal.pone.0120731

**Published:** 2015-03-23

**Authors:** Philemon Tsang, Aaron Z. Bailey, Aimee J. Nelson

**Affiliations:** Department of Kinesiology, McMaster University, Hamilton, Canada; University of Ottawa, CANADA

## Abstract

Rapid-rate paired associative stimulation (rPAS) involves repeat pairing of peripheral nerve stimulation and Transcranial magnetic stimulation (TMS) pulses at a 5 Hz frequency. RPAS over primary motor cortex (M1) operates with spike-timing dependent plasticity such that increases in corticospinal excitability occur when the nerve and TMS pulse temporally coincide in cortex. The present study investigates the effects of rPAS over primary somatosensory cortex (SI) which has not been performed to date. In a series of experiments, rPAS was delivered over SI and M1 at varying timing intervals between the nerve and TMS pulse based on the latency of the N20 somatosensory evoked potential (SEP) component within each participant (intervals for SI-rPAS: N20, N20-2.5 ms, N20 + 2.5 ms, intervals for M1-rPAS: N20, N20+5 ms). Changes in SI physiology were measured via SEPs (N20, P25, N20-P25) and SEP paired-pulse inhibition, and changes in M1 physiology were measured with motor evoked potentials and short-latency afferent inhibition. Measures were obtained before rPAS and at 5, 25 and 45 minutes following stimulation. Results indicate that paired-pulse inhibition and short-latency afferent inhibition were reduced only when the SI-rPAS nerve-TMS timing interval was set to N20-2.5 ms. SI-rPAS over SI also led to remote effects on motor physiology over a wider range of nerve-TMS intervals (N20-2.5 ms – N20+2.5 ms) during which motor evoked potentials were increased. M1-rPAS increased motor evoked potentials and reduced short-latency afferent inhibition as previously reported. These data provide evidence that, similar to M1, rPAS over SI is spike-timing dependent and is capable of exerting changes in SI and M1 physiology.

## INTRODUCTION

Much of our understanding of somatosensory-motor interactions originates from seminal studies in monkeys that capitalized on the propensity for short and long-term plasticity in the primary somatosensory cortex (SI). Following disruption to the postcentral gyrus in macaque monkeys, these studies revealed changes in the response properties of motor cortical and spinal neurons [1], or in motor performance [2]. In humans, SI demonstrates rapid homosynaptic plasticity using methods including tactile co-activation [3] and repetitive transcranial magnetic stimulation (rTMS) and can therefore be used as a model to investigate the influence of somatosensory processing in the control of movement. For example, rTMS delivered using continuous theta-burst stimulation (cTBS) over SI decreases somatosensory evoked potentials (SEPs) [4], increases corticospinal excitability [5,6] and reduces short-latency afferent inhibition (SAI) [6], a neural circuit implicated in sensorimotor integration [7]. Collectively, these data indicate that homosynaptic plasticity within SI leads to changes within SI itself and acts remotely to alter motor cortical physiology.

In humans, heterosynaptic plasticity is evoked by paired associative stimulation (PAS) whereby repeat pairing of single TMS pulses over SI and peripheral nerve stimulation are timed to promote coincidental or non-coincidental arrival at a neuronal population. PAS promoting coincident input arrival in SI increases SEPs [8], albeit changes in SEPs are not always present [9,10]. Further, PAS over SI does not appear to alter motor cortical physiology such that corticospinal excitability is unchanged [9]. PAS requires ∼ 10–30 minutes for delivery of 200–600 nerve-TMS pulses and the after-effects are influenced by attention [11], arousal [12] and cortisol levels [13]. A faster approach to PAS is called rapid-rate PAS (rPAS) whereby 600 pulses at 5 Hz are delivered over 2 minutes [14]. RPAS over the primary motor cortex (M1) increases motor evoked potentials (MEPs) [14,15] and reduces SAI [14,15] for up to one hour following stimulation [14,15]. M1-rPAS exhibits spike-timing dependency since LTP-like effects only occur when the afferent volley and polysynaptic effects elicited by TMS are timed for their coincident arrival in M1 [14].

In the present series of experiments, we deliver rPAS to SI and examine effects on SI and M1 physiology. SI physiology was measured using the early SEP components (N20, P25, N20-P25) and SEP paired-pulse inhibition (PPI). M1 physiology was measured using MEPs and the SAI sensorimotor circuit. Our results confirm previous findings of M1-rPAS [14] with increased MEP and decreased SAI for up to one hour following stimulation. Our novel findings indicate that SI-rPAS operates in a narrow time window in which SEP PPI is reduced, MEPs are facilitated and SAI is reduced. These data indicate that short-term heterosynaptic plasticity induced by SI-rPAS is spike-timing dependent and is coincident with changes in sensorimotor integration.

## METHODS

### Participants

Twelve individuals participated in the study of which twelve (4 Males, Mean age = 20.9 ± 2.9), seven (3 Males, Mean age = 21.6 ± 3.3), eight (2 Males, Mean age = 21.6 ± 3.3) and ten (3 Males, Mean age = 21 ± 3.1) took part in Experiments 1 through 4, respectively. All participants were right-handed as determined by a subset of the Edinburgh Handedness Scale [16].

### Ethics Statement

This study was approved by the McMaster Research Ethics Board and conformed to the Declaration of Helsinki. Written informed consent was provided by each participant.

### Electromyography (EMG) Recording

Surface electrodes (9 mm diameter Ag-AgCl) were used to record electromyography (EMG) from the abductor pollicus brevis (APB) muscle of the right hand with a tendon-belly arrangement. EMG recordings were band-passed filtered between 20 Hz and 2.5 KHz, amplified x1000 (Intronix Technologies Corporation Model 2024F with Signal Conditioning; Intronix Technologies Corporation, Bolton, Ontario, Canada) and digitized at 5 KHz by an analog-to-digital interface (Power1404; Cambridge Electronics Design, Cambridge, UK).

### Neuronavigation and Single-Pulse Transcranial Magnetic Stimulation (TMS)

Single-pulse TMS was applied using a custom-built 50 mm diameter figure-of-eight branding coil connected to a Magstim 200^2^ stimulator (Magstim, Whitland, UK). This coil was used for measures of MEPs and SAI in all experiments. The motor hotspot for APB was identified as the optimal location to elicit MEPs with the lowest intensity and most consistent response with the coil positioned ∼ 45° to the mid-sagittal plane to induce a posterior-to-anterior monophasic current in the cortex. The motor hotspot was marked by digital registration using a standard MRI template via Brainsight 2 Neuronavigation (Rogue Research, Canada). The motor hotspot was verified across Experiments and adjusted if necessary.

### Somatosensory evoked potentials (SEPs)

SEPs were recorded over left-hemisphere SI following electrical stimulation of the right median nerve (square wave pulse, 0.2 ms duration, 3 Hz) using a surface bar electrode with the cathode positioned proximal (Grass SD 9, Grass Technologies, West Warwick, USA). Median nerve stimulation intensity was set to the minimum intensity to elicit a visible twitch of the thumb (i.e. motor threshold, MT). The active scalp electrode was placed at electrode position C3’ located 2 cm posterior to C3 and referenced to Fz (International 10–20 System) with the ground placed over the left clavicle [17]. EEG recordings were amplified (x 10 K) and filtered from 2–2500 Hz (Intronix Technologies Corporation Model 2024F with Signal Conditioning, Bolton, Canada). Electrode impedances were maintained at < 5 kΩ (UFI Checktrode, Model 1089 Mk III, UFI, Morro Bay, USA). Each SEP trace was the time-locked average of 500 stimuli. The N20 that represents the arrival of somatosensory afference to area 3b [18] was used to adjust the ISI for SAI for each subject. To assess changes in SI intracortical inhibition, PPI was elicited by paired stimulation of the median nerve (MT, 0.2 ms) delivered with an ISI of 30 ms [19].

### Rapid-Rate Paired Associative Stimulation (rPAS)

RPAS was applied using a Magstim Super Rapid^2^ stimulator (Magstim, Whitland, Dyfed, UK) connected to a figure-of-eight air cooled coil with the handle pointed 45 degrees to the mid-sagittal plane to induce the first current in the cortex in the posterior-to-anterior direction. Using this coil, resting motor threshold (RMT) was determined at the motor hotspot in 1% increments of the maximum stimulator output (MSO). RMT was defined as the minimum stimulation required to evoke MEPs with amplitude ≥ 50 μV in 5 out of 10 consecutive trials [20]. For nerve stimuli, sensory threshold (ST) was defined as the minimum intensity (volts) at which the subject reported sensation following nerve stimulation in 2 out of 4 consecutive trials [7,14]. Intensities were tested in increments of 2V. RPAS was delivered with 600 pairs of median nerve stimuli (2 x ST, 0.5 ms) and single TMS pulses (70% RMT) at 5 Hz over APB hotspot (for M1-rPAS) [1] and at 2 cm posterior to the APB hotspot (for SI-rPAS) and measured using Brainsight 2 Neuronavigation [21–23].

### Motor Evoked Potentials and Short-Latency Afferent Inhibition

MEPs were collected by averaging the response of 15 single TMS pulses over M1 at an intensity to evoke MEPs of ∼ 1 mV peak-to-peak amplitude in APB. The intensity to evoke ∼ 1 mV MEP amplitude was determined prior to the pre-rPAS block and was held constant throughout the session.

For SAI, the TMS intensity was set to elicit MEP amplitudes of ∼ 1 mV. Median nerve stimulation was set to MT. These stimulation intensities were determined prior to baseline and were adjusted if necessary prior to each time block. The ISI between the nerve and TMS pulse were derived from the N20 component of the SEP for each individual [24]. For SAI, fifteen trials were presented randomly for each of conditioned MEP (i.e. nerve-TMS) and unconditioned MEP (TMS only) for a total of thirty trials in each block.

### Experiment Timelines

**Fig 1:**
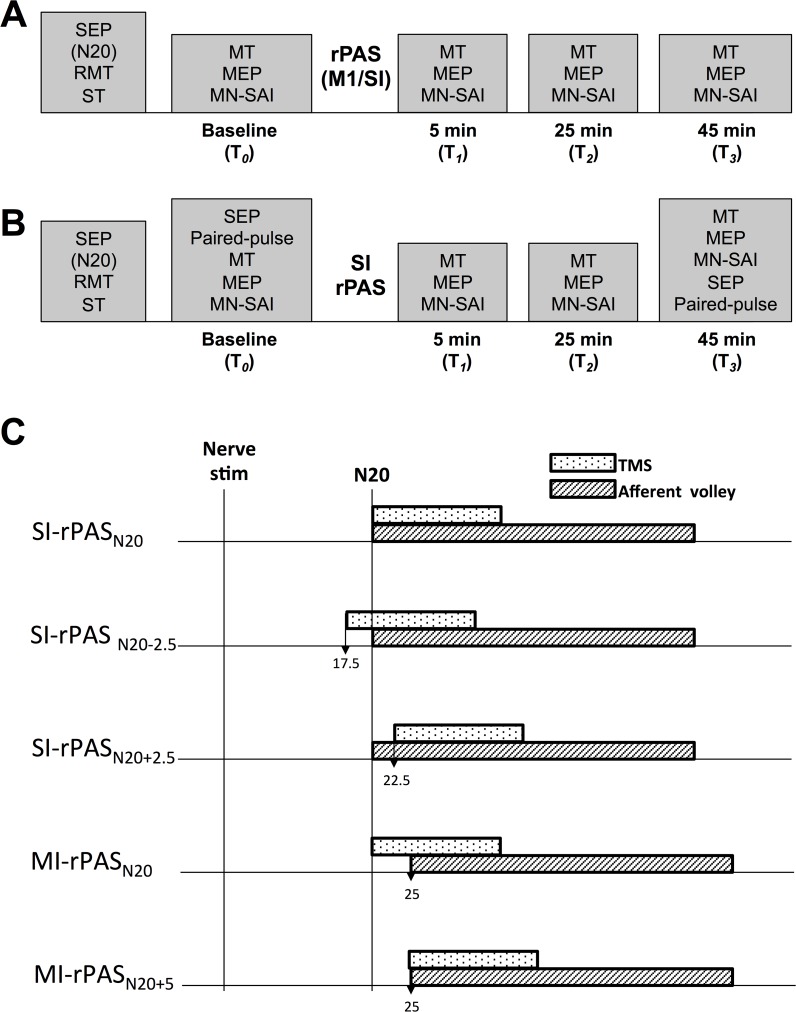
Timeline for Experiments. **A) Experiments 1 and 4**. RPAS was delivered to M1 and SI at various ISIs. Measures of MEPs and SAI were acquired from right APB before (T_*0*_) and at 5–20 minutes (T_*1*_), 25–40 minutes (T_*2*_) and 45–60 minutes (T_*3*_) following rPAS delivery. The motor threshold (MT) for nerve stimulation was determined prior to baseline and was re-evaluated before each time block **B) Experiments 2 and 3**. RPAS was delivered to SI at two different ISIs in eight participants. In addition to MEPs and SAI, SEPs and paired-pulse inhibition were acquired at the start and very end of the experiment as well. **C)** Time course showing the arrival of the afferent information (hashed bar) and TMS (dotted bar) to the location of TMS delivery (SI/M1). Note the nerve stimulation is at time 0, and when stimulating in SI the arrival of the afferent input is 20 ms, and when stimulating M1 the arrival of the afferent input is 25 ms, allowing 5 ms to travel from SI to M1 [25]. The timing of TMS stimulation was adjusted around the arrival of the afferent input (N20). TMS bar is scaled to represent 10 ms, the immediate effects of TMS within the cortex [26,27] and the afferent volley bar is scaled to represent 25 ms, the amount of time the afferent information remains in the cortex [28].

The timelines for each experiment are shown in Fig. 1A and 1B. Each session was divided into four time blocks: T_*0*_ (baseline), T_*1*_ (5–20 minutes), T_*2*_ (25–40 minutes) and T_*3*_ (45–60 minutes).

### Experiment 1: MEPs and SAI following M1-rPAS_N20+5_ and SI-rPAS_N20_

Individuals participated in two sessions (M1-rPAS, SI-rPAS) separated by a minimum of one week with the order counterbalanced across participants. Data were collected as outlined in Fig. 1A. SI-rPAS was delivered at the N20 latency for each individual (i.e. SI-rPAS_N20_) since the N20 represents the arrival of afferent input to area 3b [18]. M1-rPAS was set to N20+5 ms (i.e. M1-rPAS_N20+5_) to account for the ∼ 5 ms transmission from SI to M1 [25]. These timing intervals were chosen to promote the maximum overlap of cortical effects induced by nerve stimulation (i.e. ∼ 25 ms) [28] and TMS (i.e. immediate effects of ∼ 10 ms) [26,27] as shown graphically in Fig. 1C.

### Experiment 2: SI rPAS at N20−2.5ms

Data were collected as outlined in Fig. 1B. SI-rPAS was delivered using a timing interval of N20−2.5 ms for each participant (i.e. SI-rPAS_N20−2.5_) which is the optimal interval for inducing SI LTP-like effects using traditional PAS [8,29]. This interval assumes that the onset of TMS effects arrives at the targeted population of neurons 2.5 ms prior to the arrival of the afferent volley in 3b (Fig. 1C). Based on the traditional PAS data, we hypothesized that the P25 component would increase [8,29] and based on 5 Hz rTMS data, hypothesized that PPI would decrease [30] following SI-rPAS.

### Experiment 3: SI-rPAS at N20+2.5ms

Data were collected as outlined in Fig. 1B. SI-rPAS was delivered using a timing interval of N20+2.5ms (i.e. SI-rPAS_N20+2.5_) when the TMS effects are anticipated to occur 2.5 ms after the arrival of the peripheral afferent volley (Fig. 1C).

### Experiment 4: M1-rPAS at N20

Data were collected as outlined in Fig. 1A. M1-rPAS was delivered using a timing interval of N20 (i.e. M1-rPAS_N20_) in which afferent input that travels via relay in SI arrives at the targeted cortex ∼ 5 ms after the TMS onset of effects.

### Data Analyses

MEP data was analyzed using area since waveforms from APB were polyphasic in nature [31,32]. MEPs were calculated as the rectified EMG area within the bin that corresponded to onset and offset of EMG activity greater than background EMG for each individual. The bin width was kept consistent for the remainder of the analysis for that experiment. For SAI, the averaged conditioned MEP area (i.e. nerve-TMS) was normalized to the averaged unconditioned MEP area (TMS only) for each participant and each time block (ie. $SAI\;=\;\frac{MEP\;(nerve-TMS)}{MEP\;(TMS)}$). Two-tailed paired t-tests tested for the presence of SAI.

For Experiment 1, MEPs and SAI were analyzed with a two-way repeated measures analysis of variance (ANOVA) using within subject factors TIME (4 levels; T_*0*_, T_*1*_, T_*2*_, T_*3*_) and SITE (2 levels; M1, SI). For Experiments 2, 3 and 4, MEPs and SAI were analyzed with one-way repeated measures ANOVAs using within subject factors TIME (4 levels; T_*0*_, T_*1*_, T_*2*_, T_*3*_). Post-hoc Tukey’s honest significant difference (HSD) test was used to identify differences among the means in the event of significance.

For SEP analysis, the N20 was calculated as the difference between the baseline EMG and the first negative inflection after the stimulus, the peak-to-peak amplitude of N20-P25 was assessed as the difference between the N20 and the subsequent positive inflection and the P25 was calculated as the difference between the N20-P25 and the N20 [8,9,33]. Paired-pulse inhibition was expressed as a ratio of the amplitudes of the second N20-P25 (i.e. A2) and the first (A1) N20-P25 peaks. *A priori* planned comparisons for changes in P25 amplitude and paired-pulse inhibition were tested using one-tailed paired *t*-tests. Pearson’s correlation coefficient (*r*) analyses were performed to examine relationships between MEP, SAI, SEP amplitudes, and PPI between T_*0*_ and T_*3*_.

SPSS software (v. 20.0; SPSS, Chicago, IL) was used for statistical analysis. For ANOVAs, the Greenhouse-Geisser method was used to correct for non-sphericity. Significance was set at p ≤ 0.05.

## RESULTS

### Experiment 1. SI-rPAS_N20_ and M1-rPAS_N20+5_

All 12 participants successfully completed the two sessions (4 males, mean age = 20.9 ± 2.9). The group-averaged TMS intensity for rPAS was 40% ± 6.1 and 38.8% ± 5.5 for M1 and SI sessions, respectively, and was not different (two-tailed paired *t*-test, *p* = 0.39). Similarly, the group-averaged nerve stimulation intensity for M1 (14.7V ± 8.9) and SI-rPAS (16.3V ± 8.0) were not different (two-tailed paired *t*-test, *p* = 0.30).

**Fig 2:**
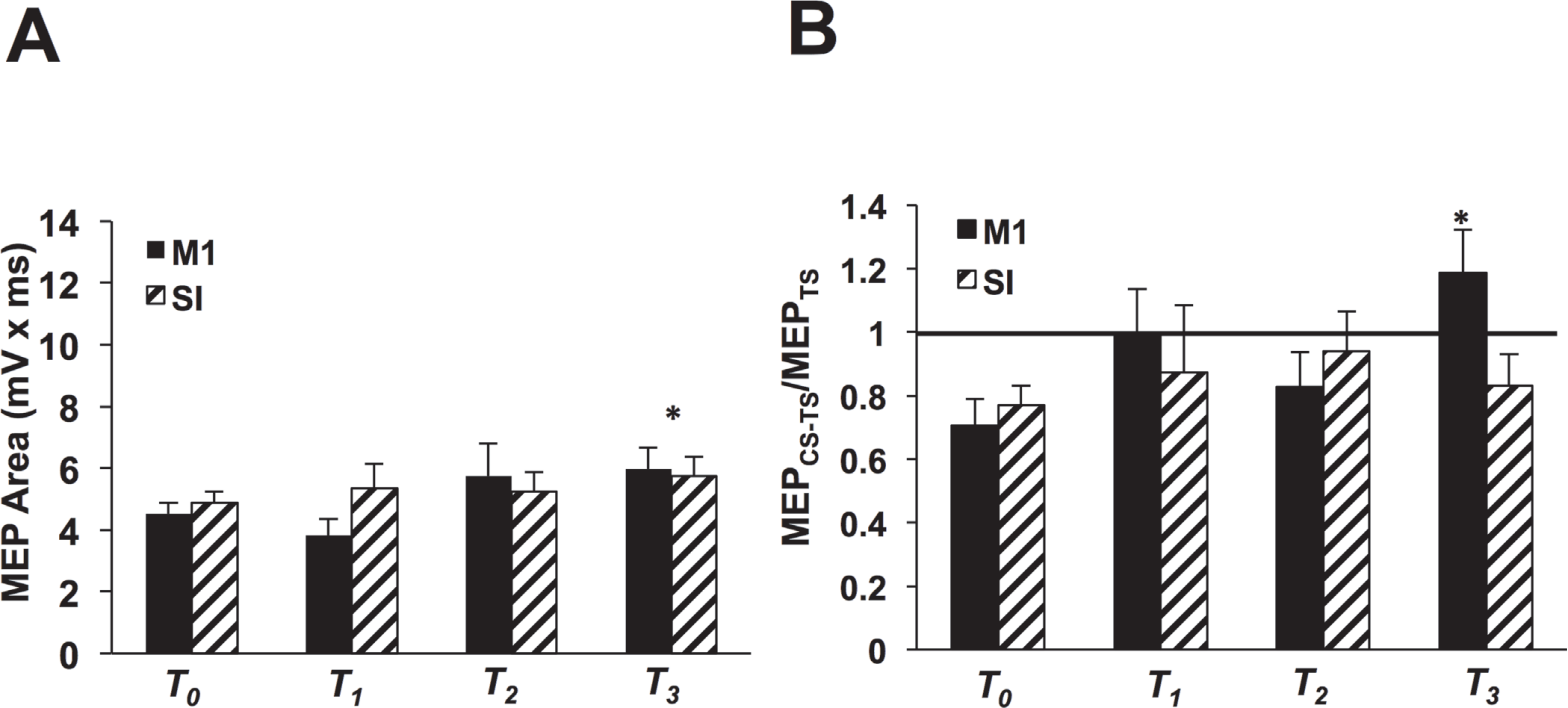
Experiment 1 with M1-rPAS_N20+5_ and SI-rPAS_N20_ (n = 12). **A)** Group-averaged MEP area with standard error of the mean following M1-rPAS_N20+5_ (solid) and SI-rPAS_N20_ (diagonal lines) at T_*0*_, T_*1*_, T_*2*_, and T_*3*_ for the right APB. Asterisk over the middle of the bars indicate that MEP area was significantly increased following M1-rPAS_N20+5_ and SI-rPAS_N20_. **B)** Group-averaged SAI with standard error of the mean following M1-rPAS (solid) and SI rPAS (diagonal lines) at T_*0*_, T_*1*_, T_*2*_, and T_*3*_ recorded from the RAPB. An asterisk over a single time block indicates where SAI was significantly reduced following M1-rPAS_N20+5_ in comparison to SI-rPAS_N20_.

At T_*0*_ MEPs were not different between M1 and SI sessions (two-tailed paired *t*-test, *p* = 0.28). Fig. 2A displays the group-averaged MEPs (with standard error of the mean) following M1-rPAS and SI-rPAS at each time block. Two-way ANOVA revealed a significant main effect of TIME (*F*_(3, 33)_ = 3.32, *p* = 0.03) without SITE (*F*_(3, 33)_ = 0.65, *p* = 0.44) or interaction effects (*F*_(3, 33)_ = 2.47, *p* = 0.08). MEPs were greater at T_*3*_ versus T_*0*_ (post-hoc Tukey’s, p < 0.05) indicating that corticospinal excitability was facilitated following rPAS to either cortical site.

Group-averaged SAI (with standard error of the mean) is shown in Fig. 2B. Two-way ANOVA revealed significant effects of TIME (*F*_(2, 22)_ = 4.64, *p* = 0.008) and TIME x SITE (*F*_(3, 33)_ = 3.99, *p* = 0.016) without a main effect of SITE (*F*_(1, 11)_ = 3.33, *p* = 0.095). Post-hoc Tukey’s HSD revealed a significant interaction at T_*3*_ where SAI was reduced following M1-rPAS but not following SI-rPAS (*p* < 0.05). At T_*3*_ following M1-rPAS, there was no difference in the conditioned versus unconditioned MEPs indicating that SAI was no longer observed (two-tailed paired *t*-tests, *p* = 0.07). In summary, M1-rPAS_N20+5_ decreases SAI and increases MEPs while SI-rPAS_N20_ increases MEPs only.

### Experiment 2. SI-rPAS_N20−2.5_

**Fig 3:**
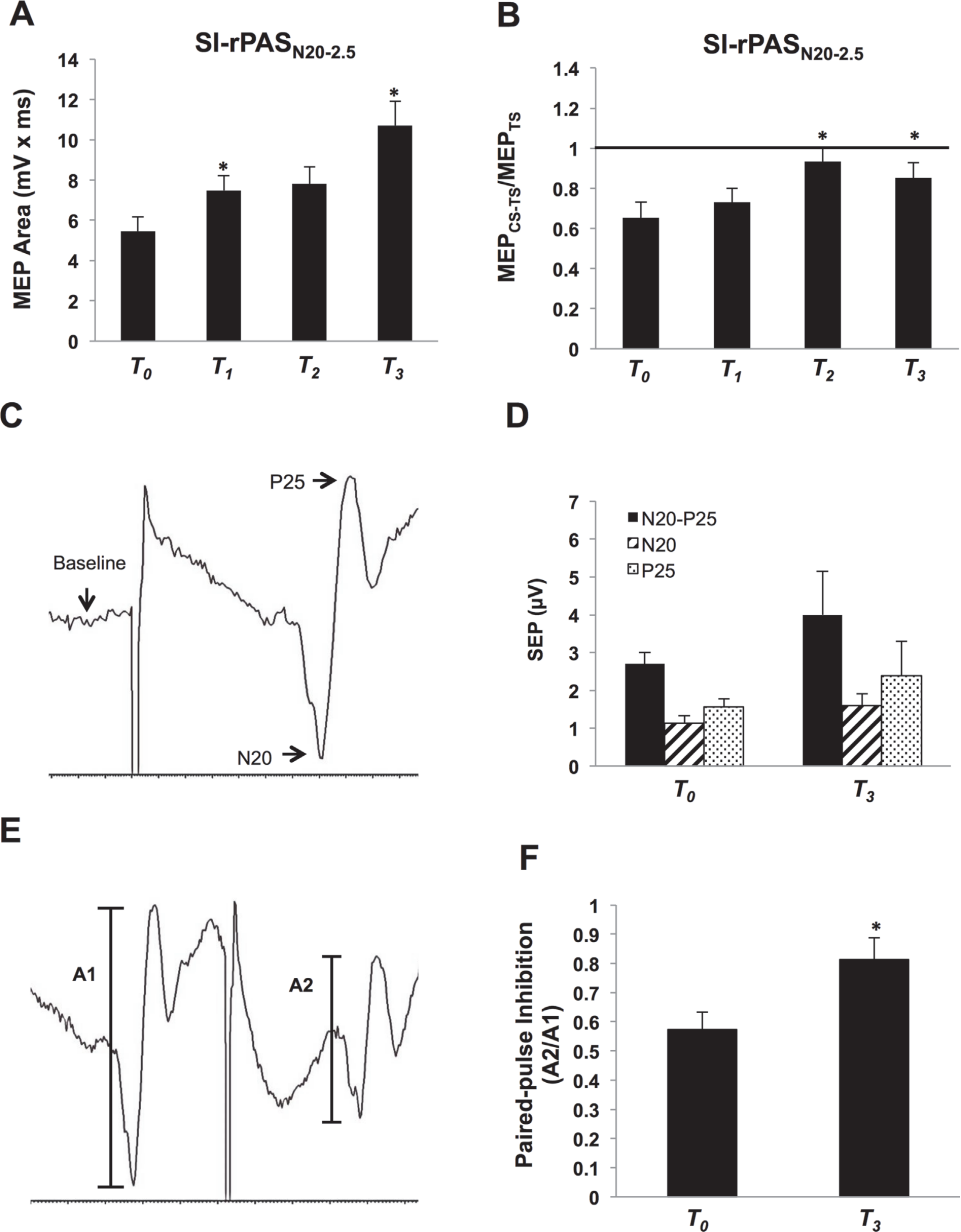
Experiment 2 with SI-rPAS_N20−2.5_ (n = 7). **A)** Group-averaged MEP area with standard error of the mean following SI-rPAS_N20−2.5_ at T_*0*_, T_*1*_, T_*2*_, and T_*3*_. Asterisk over the middle of the bars indicate that MEP area was significantly increased following SI-rPAS_N20−2.5_. **B)** Group-averaged SAI with standard error of the mean following SI-rPAS_N20−2.5_ at T_*0*_, T_*1*_, T_*2*_, and T_*3*_ recorded from the RAPB. Asterisk over a single time block indicates SAI was significantly reduced following SI-rPAS_N20−2.5_. **C)** Example SEP traces (500 epochs) from one participant before the application of rPAS; N20, P25, and N20-P25 components recorded from C3’. **D)** Amplitude of SEP components recorded at T_0_ and T_3_. **E)** Example paired-pulse inhibition traces (500 epochs) from one participant before the application of rPAS recorded from C3’. **F)** Paired-pulse inhibition measured by normalizing the later N20-P25 potential (A2) by the earlier N20-P25 potential (A1) at T_0_ and T_3_.

Seven individuals participated (3 males, mean age = 21.6 ± 3.3). The average MSO for rPAS delivery was 41.5% ± 8.0 and the average nerve stimulation for rPAS was 17.8V ± 5.5. For MEPs, one-way repeated measures ANOVA revealed a significant effect of TIME (*F*_(3, 18)_ = 8.01, *p* = 0.001) (Fig. 3A). Post-hoc Tukey’s HSD revealed increases in MEPs at T_*1*_ and T_*3*_ compared to T_*0*_ (p < 0.05).

For SAI, shown in Fig. 3B, one-way ANOVA revealed a significant effect of TIME (*F*_(3, 18)_ = 4.59, *p* = 0.015) whereby SAI was significantly reduced at T_*2*_ and T_*3*_ compared to T_*0*_ (Post-hoc Tukey’s HSD, p < 0.05). At these time blocks, there were no differences in the conditioned versus unconditioned MEPs indicating that SAI was no longer observed (T_*2*_: two-tailed paired *t*-tests, *p* = 0.40; T_*3*_: two-tailed paired t-test, *p* = 0.13).

**Fig 4:**
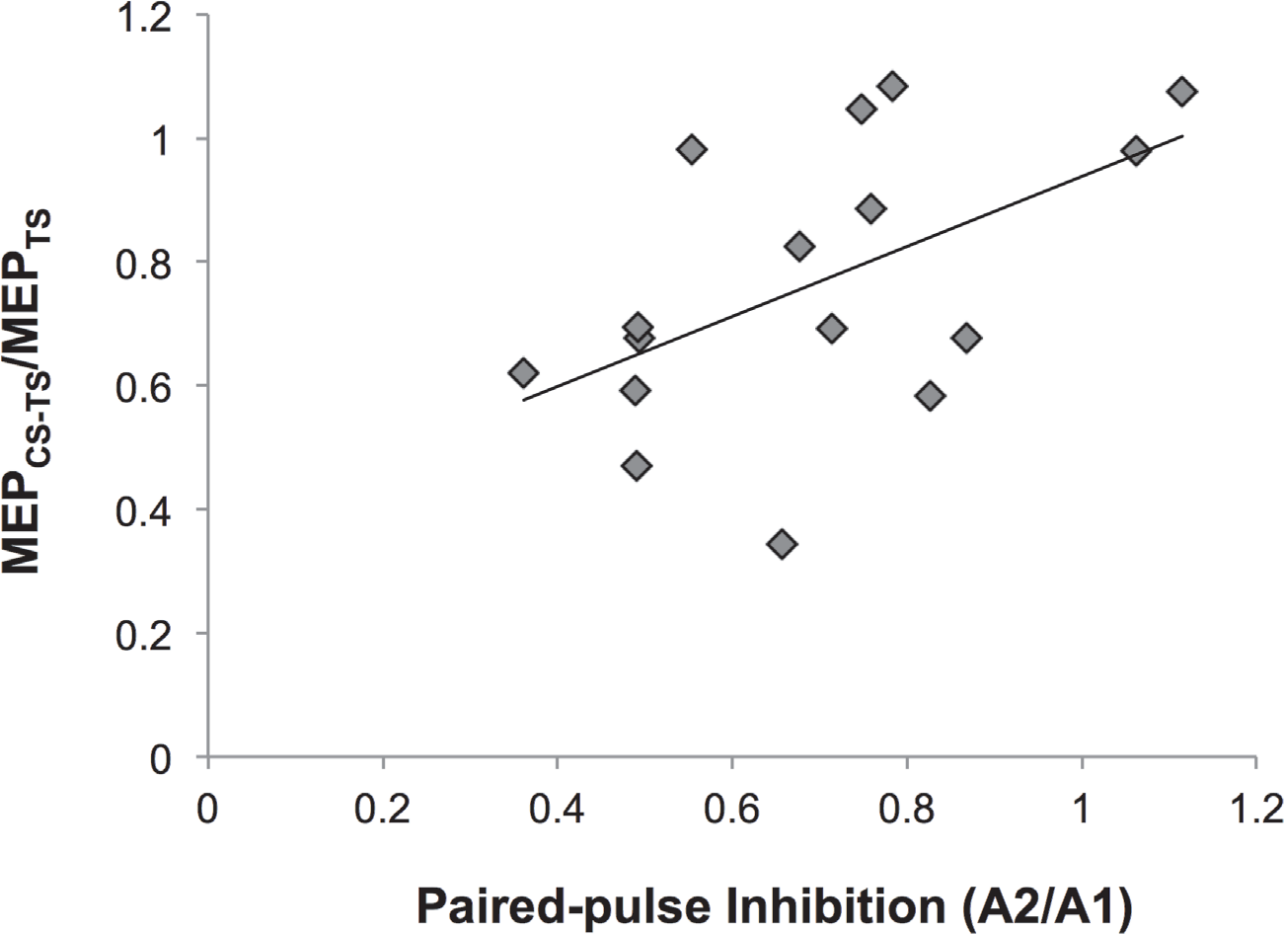
Correlation between SAI MEP ratio and paired pulse inhibition. Size of the MEP ratio was positively correlated with the PPI at T_*3*_ (r = 0.53, p = 0.03), as SAI decreases PPI decreases as well.

Fig. 3C plots an example of one individual’s SEP traces averaged over 500 epochs providing an example of the components measured. Group-averaged SEP components are shown in Fig. 3D. Comparing SEP components at T_*0*_ versus T_*3*_, the N20 (two-tailed paired t-test, p = 0.07), the N20-P25 (two-tailed paired t-test, p = 0.11) and the P25 (one-tailed paired t-test, p = 0.16) were not significantly different. Paired-pulse SEPs are shown for one participant in Fig. 3E with group-averaged data in 3F. These data revealed a significant reduction of PPI following SI-rPAS (p = 0.029). Further, SAI significantly correlated with PPI measured at T_*0*_ and T_*3*_ (r = 0.53, p = 0.03) (Fig. 4). No other correlations were observed. In summary, SI-rPAS_N20−2.5_ increases MEPs, reduces SAI, and decreases in PPI.

### Experiment 3. SI-rPAS_N20+2.5_

**Fig 5:**
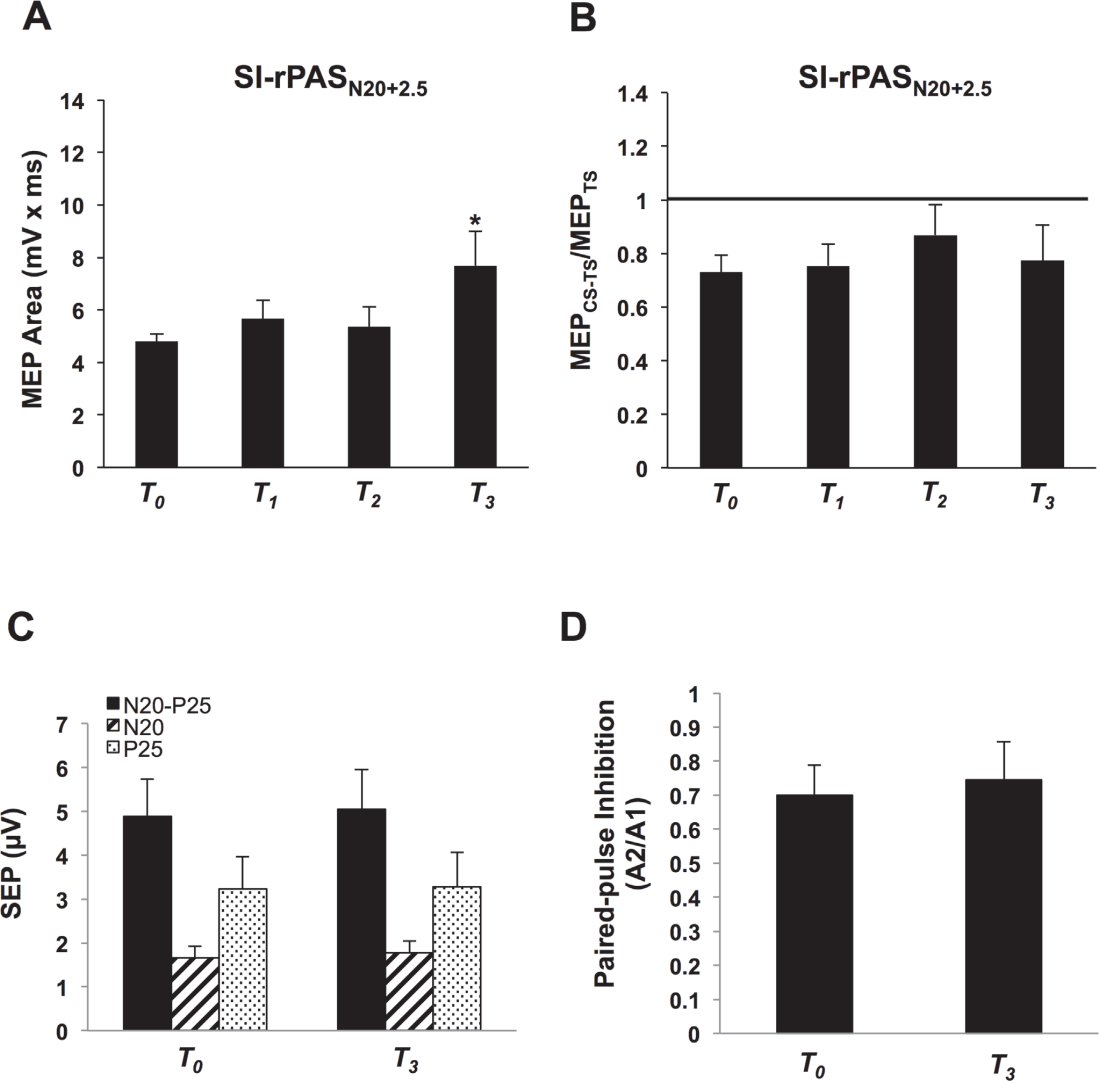
Experiment 3 with SI-rPAS_N20+2.5_ (n = 8). **A)** Group-averaged MEP area with standard error of the mean following SI-rPAS_N20+2.5_ at T_*0*_, T_*1*_, T_*2*_, and T_*3*_. Asterisk over the middle of the bars indicate that MEP area was significantly increased following SI-rPAS_N20+2.5_ at T_*3*_. **B)** Group-averaged SAI with standard error of the mean following SI-rPAS_N20+2.5_ at T_*0*_, T_*1*_, T_*2*_, and T_*3*_ recorded from the RAPB. SAI was not significantly reduced following SI-rPAS_N20+2.5_. **C)** Amplitude of SEP components recorded at T_0_ and T_3_. **D)** Paired-pulse inhibition measured by normalizing the later N20-P25 potential (A2) by the earlier N20-P25 potential (A1) at T_0_ and T_3_.

Eight individuals participated (2 Males, Mean age = 21.6 ± 3.3). The average MSO for rPAS delivery was 38% ± 6.1 and the average nerve stimulation for rPAS was 13.6V ± 9.6. For MEPs, one-way ANOVA revealed a significant main effect of TIME (*F*_(3, 21)_ = 3.97, *p* = 0.02) (Fig. 5A) with greater MEPs at T_*3*_ compared to T_*0*_ (post-hoc Tukey’s, p < 0.05). For SAI, one-way ANOVA revealed no main effect of TIME (*F*_(1.9, 13)_ = 0.55, *p* = 0.58). Fig. 5B displays group-averaged SAI (with standard error of the mean) following SI-rPAS_N20+2.5_.

SEP components were not significantly different at T_*0*_ versus T_*3*_ (Fig. 5C): N20 (two-tailed paired t-test, p = 0.38), N20-P25 (two-tailed paired t-test, p = 0.48), P25 (one-tailed paired t-test, p = 0.43). Similarly, paired-pulse SEPs were unchanged following rPAS (one-tailed paired t-test, p = 0.23) as shown in Fig. 5D. In summary SI-rPAS_N20+2.5_ increases MEPs but does not significantly alter SAI or SEPs.

### Experiment 4. M1-rPAS_N20_

**Fig 6:**
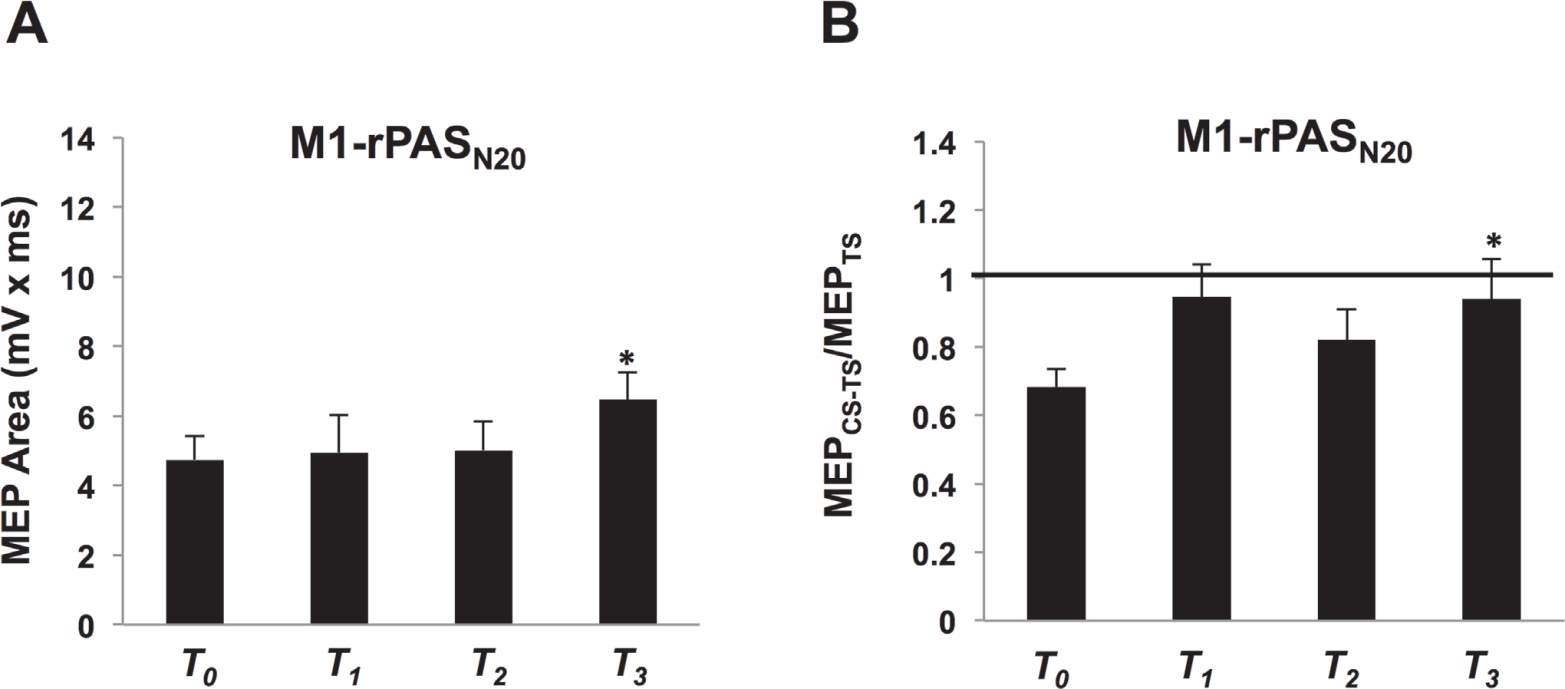
Experiment 4 with M1-rPAS_N20_ (n = 10). **A)** Group-averaged MEP area with standard error of the mean following M1-rPAS_N20_ at T_*0*_, T_*1*_, T_*2*_, and T_*3*_. Asterisk over the middle of the bars indicate that MEP area was significantly increased following M1-rPAS_N20_. **B)** Group-averaged SAI with standard error of the mean following M1-rPAS_N20_ at T_*0*_, T_*1*_, T_*2*_, and T_*3*_ recorded from the RAPB. An asterisk over a single time block indicates where SAI was significantly reduced following M1-rPAS_N20_.

Ten individuals participated (3 Males, Mean age = 21 ± 3.1), all of who were tested in Experiment 1. The average maximal TMS output for rPAS delivery was 39.7% ± 5.9 and the average nerve stimulation for rPAS was 20.5V ± 3.9. For MEPs, one-way ANOVA revealed no effect of TIME (*F*_(1.4, 12.3)_ = 1.57, *p* = 0.24) (Fig. 6A). However, *a priori* hypothesis testing, based on Experiment 1 results, revealed a significant increase in MEP area at T_*3*_ compared to T_*0*_ (one-tailed paired t-test, p = 0.03). For SAI, one-way ANOVA revealed no effect of TIME (*F*_(3, 27)_ = 2.51, *p* = 0.08) (Fig. 6B). *A priori* hypothesis testing revealed a significant decrease in SAI at T_*3*_ compared to T_*0*_ (one-tailed paired t-test, p = 0.046), similar to the results of Experiment 1. For SAI at T_*3*_ following M1- rPAS_N20_, there was no significant difference between the conditioned and unconditioned MEPs (two-tailed paired *t*-tests, *p* = 0.35).

## DISCUSSION

In the present study, we investigated rPAS targeting SI and M1. Our data supports previous reports of M1-rPAS [14,15] and makes several novel contributions. First, SI-rPAS demonstrates evidence of spike-timing dependent plasticity with changes in SI physiology occurring only at N20−2.5 ms suggesting a narrow temporal window for inducing plasticity. It is this temporal interval that also leads to reductions in the SAI sensorimotor circuit suggesting a relationship between these two measures. Second, SI-rPAS is a powerful modulator of corticospinal output over a wider temporal interval. We discuss these findings and provide possible neural mechanisms to explain the effects of SI-rPAS.

### M1-rPAS

Two previous studies examined the effects of M1-rPAS on motor cortical physiology. M1-rPAS_25ms_ increased MEP amplitude immediately and for up to 60 minutes [14,15] or facilitated MEPs beginning at ∼ 30 minutes [15]. In support of these reports, our data revealed a facilitation of MEPs that emerged at 45–60 minutes following M1-rPAS_N20+5_, a difference that may relate to the TMS intensity used (90% AMT [1] versus 70% RMT used here). Likewise, this difference may relate to our usage of RMT rather than AMT (i.e. 100% RMT translates to ∼120% AMT [34]. It is notable that MEP facilitation is similar between studies (∼ 38% versus ∼ 33% seen here) while SAI reduction was greater in the present report (∼ 48% versus ∼ 71% seen here) suggesting that an individualized approach to the N20 may be optimal for altering SAI.

The mechanisms by which M1-rPAS alters motor cortical physiology remain elusive. We tested two timing intervals for M1-rPAS (Fig. 1C) and observed greater changes in MEP facilitation (∼ 5%) and SAI reduction (∼ 33%) using the N20+5 ms versus the N20 interval. It remains unclear whether these changes are induced by the afferent volley proceeding directly to M1 (i.e. direct thalamocortical input as suggested by human intracerebral recordings) [35] or by inputs arriving through cortico-cortical connections via SI (estimated to require an additional 5 ms) [25]. Similar to our findings, traditional M1-PAS shows greatest MEP facilitation using a timing interval of 25 versus 20 ms between the nerve and TMS pulse [36]. The collective data suggests that an ISI closest to the individual’s N20 latency with an additional 5 ms is ideal for promoting the temporal coincidence of the afferent volley and the TMS induced effects in traditional M1-PAS and M1-rPAS.

### SI-rPAS

SI-rPAS was effective at inducing physiological changes in SI when delivered using a very narrow temporal window between the nerve and TMS stimuli. SI-rPAS_N20−2.5_ reduced PPI by ∼ 22% at ∼ 45–60 minutes following stimulation. A similar range of PPI reduction (21% to 56%) follows homosynaptic protocols including iTBS [3], 5 Hz rTMS over SI [30] and tactile co-activation, which also resulted in a concomitant improvement in tactile acuity [3]. Our data also suggests an emerging trend for increases in SEP components N20, P25 and N20-P25 though our sample may have been underpowered to reveal the subtle effects observed in traditional SI-PAS (n = 35 used in [8]). However, our PPI data clearly indicate that SI-rPAS is dependent upon the temporal relationship between the arrival of the afferent volley and TMS induced cortical effects; only SI-rPAS _N20−2.5_ altered SI physiology. It is notable that traditional SI-PAS only changes EEG scalp topography in SI at the N20−2.5ms ISI [29]. We also note that concomitant changes in SAI occur at N20−2.5ms such that SAI is reduced by 43% at 25–40 minutes following stimulation. Further, reduced PPI was significantly correlated with reduced SAI, suggesting that PPI and SAI may have overlapping neural mechanisms; the modulation of SI intracortical inhibition may also mediate the reduction in afferent-induced inhibition.

SI-rPAS consistently led to MEP facilitation by 45–60 minutes following stimulation. Though the neural underpinnings following SI-rPAS are not completely clear, facilitation of corticospinal excitability following SI-rPAS occurs in a generous temporal window (N20−2.5 to +2.5 ms). One possibility is that SI modulation of corticospinal excitability occurs through a different circuitry than that of PPI and SAI modulation, though it should not be ruled out that these circuits might operate through the same pathway. However, it was specifically SI-rPAS_N20−2.5_ that led to the earliest (i.e. 5–20 minutes following rPAS) and greatest MEP facilitation. Therefore, although SI-rPAS facilitates corticospinal excitability in a generous temporal window, a precise timing of N20−2.5ms allows for the greatest effects on corticospinal output and concomitant changes in PPI and SAI.

### What mechanisms may mediate SI-rPAS_N20−2.5_ changes?

The altered physiology seen after SI-rPAS_N20−2.5_ may be explained by a decrease (i.e. LTD) in the excitatory synapse between spiny stellates and layer II/III pyramidal cells. The TMS pulse provides postsynaptic depolarization of the layer II/III pyramidal cells slightly in advance of the afferent volley arrival (∼ 2.5 ms, Fig. 1C), a sequence of activation that lends itself to LTD [36]. LTD at these excitatory stellate-pyramidal synapses reduces the initial excitation of the layer II/III pyramidal cells, which thereby decreases the magnitude and duration of subsequent in-field and lateral inhibition of the pyramidal cell [28,37]. Reduced columnar inhibition may therefore present as decreases in PPI and possibly increases in SEP components (i.e. reduced gating of SEPs). Our suggestion is further supported by the finding that PPI is intensity dependent; increases in SI excitation via an increase in nerve stimulation intensity leads to increases in PPI [33,38,39]. Further, reductions in the excitation of the pyramidal cell may remotely alter M1 through long-range lateral connections from SI [40] leading to increased MEPs. Such long-range lateral projections from SI to M1 appear to have a net inhibitory influence on M1 in monkey studies; M1 excitability is decreased following cooling of SI [1]. Following SI-rPAS_N20−2.5_, inhibitory input from SI to M1 may be reduced due to a decreased firing of layer II/III pyramidal cells (similar to cooling). In humans, the SAI circuit involves inhibition of the I3 generating neuron [24,41]. Therefore, a reduction in the inhibitory influence from SI on M1 would result in disinhibition of the I3 generating neurons; decreasing SAI and increasing MEPs [6].

### rPAS M1 vs SI

As our data shows, rPAS induces plasticity in M1 and SI and similar changes to MEP and SAI are observed, albeit the magnitudes of effects are different (MEPs: 85% increase for SI-rPAS and 33% increase for M1-rPAS; similar magnitude for SAI) and the time course is different between loci (SI-rPAS modulates MEPs/SAI earlier than M1-rPAS). One important difference between loci is the ISI necessary to achieve maximal changes to MEPs and SAI (N20−2.5ms for SI-rPAS and N20+5ms for M1-rPAS). This difference of 7.5 ms is consistent with modelling and empirical findings from past reports testing traditional PAS [8,42]. Muller-Dahlhaus et al. (2010) speculate that the difference in ISIs between the two sites might be due to the time needed for the afferent input to propagate through SI and arrive in M1, where it is able to coincide with the TMS input on a common neuronal population [42]. Indeed, our results support an LTP like effect when M1-rPAS was applied at an ISI of N20+5 ms which allowed for the presynaptic afferent volley to precede or coincide with the postsynaptic TMS induced effects. However, alternative to Muller-Dahlhaus et al. (2010), we propose that the present findings in SI at N20−2.5 ms suggest LTD at excitatory synapses, which reduces the lateral/in-field inhibition thus decreasing PPI and SAI while increasing MEPs.

### Limitations

A limitation in the present study are influences that contribute to efficacy of heterosynaptic stimulation such as age [43], attention [11] and time of day [44]. However, we used a much shorter protocol to circumvent the issue of attention. Further, subjects were also instructed to attend to a fixation point on the wall while rPAS was applied. The time (AM vs. PM) in which testing was performed was kept consistent within each participant. A second limitation we cannot eliminate is the possibility that rPAS evoked excitability changes at the spinal level, however, SAI is considered a cortically mediated circuit [24,45] this possibility is unlikely. Also, 70% RMT is unlikely to generate any descending volleys [27]. Further, although tactile psychophysics were not measured in the present study, perception may be altered following TMS plasticity protocols targeting SI. Future studies should investigate the effects of SI-rPAS on spatial and temporal tactile perception [21,46]. Finally, the longevity of the SI-rPAS effect remains unclear. M1-rPAS has effects that persist for up to 6 hours following stimulation [14].

### Summary

In summary, the application of rapid-rate heterosynaptic stimulation targeting SI led to long-lasting changes in SI excitability and remote changes in M1, supporting of the potent SI influence of motor physiology. Further investigation and optimization of these protocols have potential as rehabilitation strategies for altering sensorimotor control.
